# Everything is everywhere but *Escherichia coli* adapts to different niches

**DOI:** 10.1093/ismejo/wraf267

**Published:** 2025-12-18

**Authors:** William Monteith, Made A Krisna, Biel Garcias, Elizabeth A Cummins, David J Kelly, Aidan J Taylor, Samuel K Sheppard

**Affiliations:** The Milner Centre of Evolution, Department of Life Sciences, University of Bath, Claverton Down, Bath BA2 7AZ, United Kingdom; Ineos Oxford Institute, Department of Biology, Life and Mind Building, University of Oxford, South Parks Road, Oxford OX1 3EL, United Kingdom; Ineos Oxford Institute, Department of Biology, Life and Mind Building, University of Oxford, South Parks Road, Oxford OX1 3EL, United Kingdom; Nuffield Department of Population Health, University of Oxford, Old Road Campus, Oxford OX3 7LF, United Kingdom; Department of Animal Health and Anatomy, Universitat Autònoma de Barcelona, 08193 Cerdanyola del Vallès, Spain and Department of Microbiology and Immunology, Institute of Biomedicine, Sahlgrenska Academy, University of Gothenburg, Gothenburg 41390, Sweden; Ineos Oxford Institute, Department of Biology, Life and Mind Building, University of Oxford, South Parks Road, Oxford OX1 3EL, United Kingdom; School of Biosciences, University of Sheffield, Sheffield S10 2TN, United Kingdom; School of Biosciences, University of Reading, Reading RG6 6UR, United Kingdom; Ineos Oxford Institute, Department of Biology, Life and Mind Building, University of Oxford, South Parks Road, Oxford OX1 3EL, United Kingdom

**Keywords:** *E. coli* population genomics, genome-wide association study (GWAS), host adaptation

## Abstract

Pathogens that are harmless in one environment can cause a serious disease in another. Among host-associated bacteria, transition between hosts can have serious consequences for animal and human health. However, much remains unknown about how adaptation shapes bacterial distribution in the wild. Here, investigating the ecological genomics of *Escherichia coli* from diverse hosts and environments, we address the idea that bacteria disperse freely, and challenge the “everything is everywhere” paradigm. Using comparative genomics and parallelised high throughout pangenome-wide association studies (900 experiments) we investigate lineage distribution and identify adaptive genomic signatures associated with host species, physiology and ecology. Our findings provide insights into bacterial niche adaptation, emphasize the impact of agriculture on microbial evolution, and inform One Health frameworks by linking genomics, host ecology, and the emergence of antimicrobial resistance.

## Introduction

Bacteria inhabit almost every environment on Earth and studying their distribution reveals the nature of life’s adaptability. Among the bacteria, *Escherichia coli* stands out as the best understood species in terms of the genetics underlying adaptation. This is largely because of decades of research as a model laboratory organism [1, 2], but little is known about how adaptation influences spatial distribution patterns in the wild: this is influenced by a combination of ecological and biogeographical factors, with the latter emphasizing how isolation drives diversification. Physical isolation has long been considered important in driving speciation, including by Darwin [3], but in microbes it has been largely perceived as unimportant due to their widespread dispersal and vast populations. This view has been summed up as “everything is everywhere, but the environment selects” [4], meaning that all microbes exist globally, but only thrive where conditions suit them. However, recent genomic studies reveal that bacteria can exhibit biogeographic patterns [5–7] and localized adaptation [8, 9], challenging the idea of universal dispersal and revealing the importance of niche adaptation.

Bacterial adaptation has been well characterized in long-term laboratory passage experiments, demonstrating the nature and rate of *E. coli* evolution in laboratory culture media [10], but adaptation is much more complex *in vivo.* When colonizing the gut of warm-blooded animals, the natural habitat of *E. coli*, bacteria face challenges linked to host immune defenses, a complex nutritional environment, and competition with other bacteria. In this natural host milieu, observing bacterial population genetic structuring can reveal adaptation at different levels of organization. For example, in populations of *Staphylococcus aureus*, different lineages are restricted to specific birds and mammal hosts [11]. Conversely, in *Campylobacter jejuni*, some lineages are common to multiple livestock bird and mammalian hosts, but are distinct from those found in wild birds [12–14]. This is consistent with adaptation occurring at both the level of host species and at a higher level of host ecology (livestock vs. wild).

In *E. coli*, there is initial support for the “everything is everywhere” hypothesis, as deep branching phylogroups can be observed on phylogenetic trees. However, although some are associated with environmental isolates or clinical infection [15], there is no clear link to host source. Adaptation to livestock and the farm environment has been described for *E. coli* [16, 17], observed as reduced *E. coli* diversity among domestic compared to wild deer, and as different metabolic capabilities among strains from wild boar compared to domestic pigs [18, 19]. Adaptation to dietary differences has been shown to be an important factor, with *E. coli* from wild boar more likely to harbor specific iron acquisition genes, but other factors are also important. As intensive livestock production increases, chronic stress and local climate have been shown to alter the microbiome of hosts [20–23], but perhaps the best example of farm niche adaptation is the spread of antimicrobial resistance (AMR). This is thought to result from selection for resistance imposed by the widespread use of antimicrobials for disease prevention, treatment, and growth promotion [24, 25].

Most *E. coli* are harmless or even beneficial [26, 27] but certain pathogenic strains cause severe illnesses in livestock and humans. Common pathologies include diarrhea, urinary tract infections, respiratory disease, bloodstream infections, and colibacillosis in livestock [15, 28, 29]. As the scale of intensive agriculture increases [30], *E. coli* are excreted into the environment on a massive scale, creating numerous pathways to enter the human food chain. For certain strains, such as *E. coli* O157, zoonotic transmission on contaminated food poses a significant risk to human health [31]. More generally, increased opportunity for host transition has potential to promote the emergence of new pathogenic lineages and the spread of AMR.

Despite extensive work on *E. coli* population genetics, there is little understanding of the distribution and adaptation of natural animal host populations. There is some evidence for host associated lineages [32], but this declines with distance and so may reflect transmission opportunity rather than true host adaptation [33–35]. It may also be the case that lineages reflect a higher organizational level such as adaptation to host gut physiology (monogastric vs. ruminant vs. bird) [36, 37], or even the broader ecology of farmed vs. wild animal niches. Here, analyzing the ecological genomics of *E. coli* isolated from various animal host species we address the pervasive, and perhaps mis-informed, “everything is everywhere” aphorism. This work improves our understanding of niche adaptation and bacterial dispersal and provides a quantitative basis for One Health frameworks.

## Material and methods

### *E. coli* isolate genomes

A total of 5259 *E. coli* genome assemblies were retrieved from public databases, including the PATRIC database [38] and PubMLST [39]. Initially, all genomes isolated from animal sources were selected and their associated metadata were downloaded and assemblies with a sequencing depth < 30 were removed. Only isolates from gastrointestinal sources were included and those from food products, such as chicken, pork or beef meat were excluded. To ensure *E. coli* taxonomy was correctly assigned, ribosomal-MLST species identification was applied to the genomes [40]. Finally, to ensure quality control, a Neighbor-Joining tree was constructed based on a MASH-generated distance matrix, incorporating sequence data from all samples, to manually remove outliers, using rapidNJ (version 2.3.2, default parameters) [41]. The assemblies of 5259 *E. coli* isolates that met these criteria were downloaded and deposited in the PubMLST *E. coli* database (Supplementary Table S1).

### Pan-genome archiving and phylogenetic reconstruction

Coding sequences were identified in each genome by automated annotation using Prokka (version 1.13; default parameters) [42]. Panaroo (version 1.2.10; moderate clean-mode) [43] was used to identify clusters of orthologous genes (COGs). COGs shared by >95% of isolates were classified as part of the core genome, and the accessory genome included all other COGs present in at least one isolate. Additional scripts provided by Panaroo were used to reannotate the gff annotation files of isolates based on gene annotations assigned by Panaroo.

The pan-genome size was predicted for isolates belonging to each source and phylogroup based on the number of unique gene clusters identified by Panaroo. The pan-genome size was predicted for a population size of 100 isolates per source. However, to account for variation in phylogenetic distance caused by biased sampling within each source, we applied the following model, proposed by Park *et al.* [44]:


$$ \log{n}_i\approx{\beta}_0+{\beta}_1\log \left({D}_i+1\right)+{\beta}_2\log{N}_i $$

where *N* is the number of genomes, *D* is the sum of branch lengths calculated from a core-genome phylogeny and *n* is the pan-genome size. The scientific computing module of Python, *scipy.optimize.curve_fit* was used to optimize parameters to fit the model with the observed values using the nonlinear least squares method. The model was applied to 100 random samples of 100 genomes per source (Supplementary Table S4).

When considering the phylogenetic distance between isolates, the PIRATE pan-genome pipeline (version 1.0.4; default parameters) [45] was used to produce a core-genome alignment (length: 3314331 base pairs) by concatenating the genes shared by >95% of isolates. The phylogenetic relationship between isolates was inferred from core-genome alignments by maximum-likelihood using RAxML (version 8.0.0; GTRGAMMA model of substitution) [46]. The maximum-likelihood phylogeny and core-genome alignment were provided as input for ClonalFrameML (version 1.12; default parameters) [47], which was used to reconstruct the phylogeny whilst masking the effect of recombination taking place within the core genome.

### Pangenome-wide association studies

Bacterial populations vary greatly in their genetic content, and we aimed to capture all the genetic variation present within the population of 5259 *E. coli* isolates, including variation in the accessory genome, and infer adaptation of *E. coli*. To achieve this, we used a unitig *k*-mer definition of sequence variation. Unitigs are variable length *k*-mers extracted from a compressed de Bruijn graph constructed from the population assemblies [48]. Multiple genome-wide association studies were performed to screen for associations across the three phenotypic classifications: host ecology, host species, and host physiology. Specifically, using elastic net regression models implemented in Pyseer (version 1.3.6) [49], we assessed the correlation of *k*-mers with nine sources: (i) livestock, companion and wild animals; (ii) pigs, cattle, and chicken; (iii) monogastric mammals, ruminants, and birds. In addition, a pairwise distance matrix derived from the phylogeny of each sample group was used to derive weighted *P* values.

To mitigate bias caused by covariates, we implemented an iterative random sampling procedure using custom python scripts. Each source underwent 100 separate GWAS experiments, each comparing 100 source-specific isolates to 100 control isolates. The selection of isolates was randomized, except for stratification by source. This approach was designed to maximize phenotypic variation in the control group and reduce the rate of false–positive associations caused by sources that are overrepresented in our dataset, but also ensured comprehensive coverage of all genetic variation present within the pan-genome.

Statistical significance was determined using a Bonferroni correction based on the average number of *k*-mers tested in each experiment to negate the influence of population structure. Significant *k*-mers were mapped to the pan-genome using the BWA Fastmap and MEM algorithms (version 0.7.17) [50]. Functional annotation of genes was automatically assigned based on sequence orthology using eggNOG-mapper (version 2.1.11) [51]. Plasmid associated genes were defined as all genes located on a plasmid sequence identified using MOB-suite [52]. Significant *k*-mers identified by GWAS underwent further association tests to determine their significance within the entire population. The specificity, sensitivity and Cramer’s V statistic were used to determine the strength of association between *k*-mers and each of the niche categories. The scientific computing module of Python, “scipy.stats.chi2_contingency” was used to calculate the chi-squared statistic and Cramer’s V was computed by taking the square root of this value, divided by the sample size and the minimum number of dimensions shared by the nominal data minus 1. Niche segregating *k*-mers included those with a specificity >60%, sensitivity >25%, and Cramer’s V >0.1 in at least one niche category.

### Source attribution model

The elastic net model, implemented in Pyseer [49] is a generalized linear regression model that can be used to predict phenotypes in new populations. For each niche category an elastic net model was generated based on the distribution of niche-segregating *k*-mers and used to predict the source of isolates in a novel dataset.

**Figure 1:**
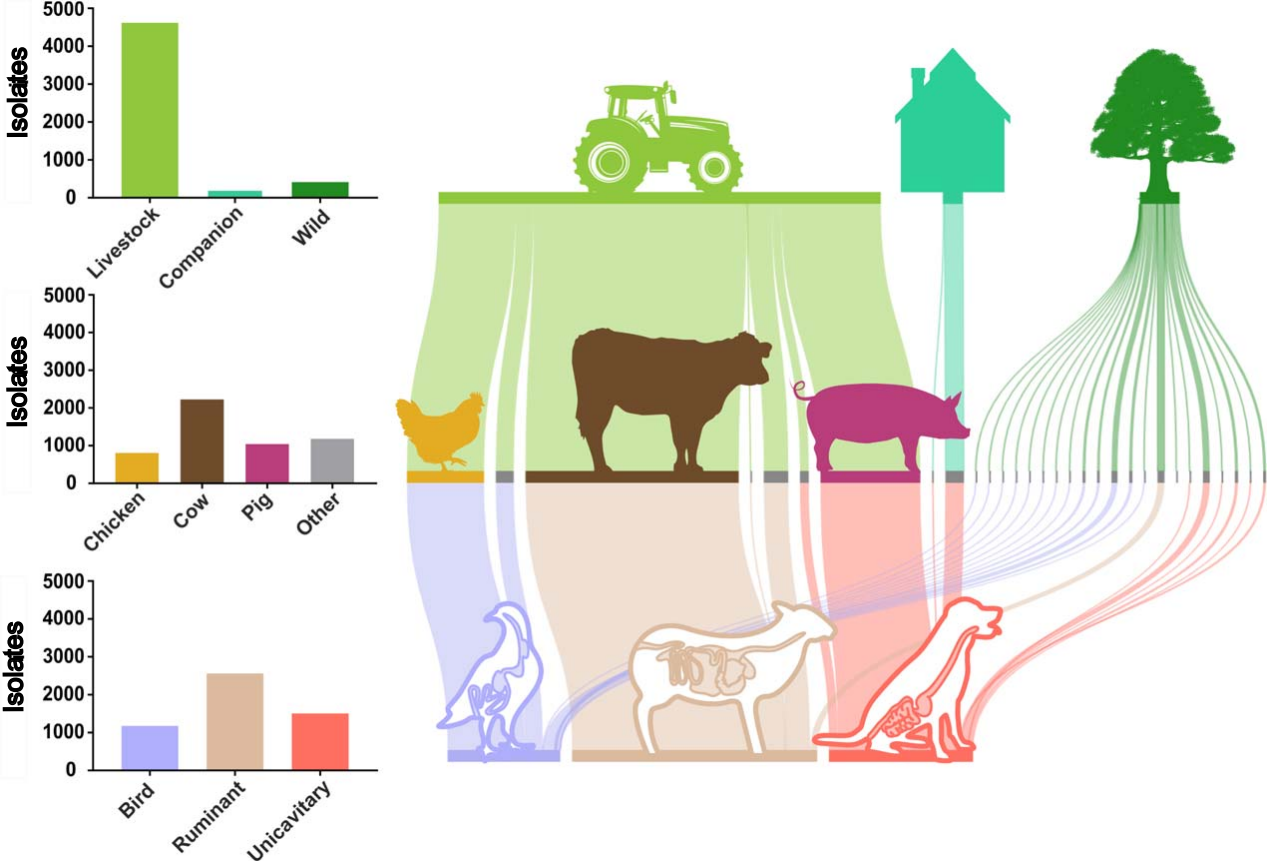
Niche organization of animal-derived *E. coli* isolates. Genomes from 5259 *E. coli* were sampled from 31 host species and 58 countries. Isolates were divided into nine source categories based on the ecology of their host organism: (A) livestock, companion, and wild; (B) chicken, cow and pig; (C) birds, ruminants and monogastric mammals.

The first stage of source attribution involved dividing the original collection of *E. coli* genomes (training dataset, *n* = 5259) into two groups; 75% of isolates were assigned to a training population and 25% of isolates were assigned to a test population. Strains were split into each group randomly except for stratification by source to ensure that all niche categories were present in both datasets. For each niche category, the presence of niche-segregating *k*-mers was used to fit an elastic net model to the training population. The fitted models were subsequently used to predict the source of isolates in the test population. As the true source of these isolates was known, sensitivity and specificity scores were calculated to quantify the ability of each model to correctly assign isolates to each niche category.

The second stage of source attribution involved re-training the models by including all isolates (*n* = 5259) in the training dataset. The prediction accuracy was assessed using a new dataset composed of previously undiscussed isolates. This validation dataset was composed of *E. coli* genomes reported in research by Tiwari *et al.* [19], and included 226, 256, and 240 isolates from chicken, cattle, and pig hosts, respectively. The short-read data for 722 isolates were downloaded using the NCBI SRA Toolkit, adapter sequences were removed using Trimmomatic (version 0.39, default parameters) [53] and draft genome assemblies were assembled using SPAdes (version: 3.14.1, default parameters) [54].

## Results

### *Escherichia coli* isolates can be assigned to three levels of niche organization

Genomes of 5259 *E. coli* isolates, collected from 31 host species across 58 countries between 1947 and 2019, were analyzed to represent the global diversity of animal-derived *E. coli* (Supplementary Table S1). *Escherichia coli* isolates were assigned at three levels of niche organization: (i) “Ecology”, livestock (*n* = 4619), companion (*n* = 223), and wild (*n* = 417); (ii) “Species”, chicken (*n* = 807), cattle (*n* = 2228), pig (*n* = 1043), and other (*n* = 1181); (iii) gut “physiology”, bird (*n* = 1178), ruminant (*n* = 2562), and monogastric mammals (*n* = 1506). This sample-phenotype structuring allowed identification of genomic factors influencing *E. coli* adaptation at three different levels (Fig. 1).

*Escherichia coli* isolates from livestock animals represent the largest ecological environment investigated, with cattle, chickens, and pigs accounting for 88% of the 4619 livestock isolates. Wild animals encompassed 22 nondomesticated species and therefore are the most diverse category. In contrast to livestock, which occupy a specific ecological niche and have little contact with other species, wild animals inhabit natural environments with minimal human intervention and complex interspecies interactions. We also included a discrete category for companion animals, including dogs and cats, which reflects their unique human-associated lifestyle, distinct from that of the other groups.

Three distinct gut morphologies were present among the host species analyzed. Ruminants, including cattle, sheep, and deer, possess four-chambered digestive systems specialized in digesting fibrous plant material through microbial fermentation. Birds process a varied diets and have unique digestive systems comprising a muscular crop, glandular stomach (proventriculus), and a specialized grinding organ (gizzard). Finally, monogastric mammals, including pigs, horses, and most other nonruminant mammals, have a simpler single-chambered stomach followed by intestines. However, even within this group gut morphology varies significantly between species, reflecting their respective dietary adaptations. Beyond physiology, ecological niche (domestic or wild) strongly influences diet and therefore the gut microbiome may vary considerably even within the same host species.

Variable geographical distribution was observed among the three levels of niche organization. For example, most *E. coli* isolated from domestic mammals originated from North America (*n* = 1527) or Asia (*n* = 1147). This bias is driven by ruminant associated samples, which predominantly originated in North America (*n* = 1270). Conversely, samples associated with monogastric mammals were of approximately equal origin between North America, Europe, Asia, and Oceania. There were only a few (*n* = 15) *E. coli* isolates from Africa, highlighting the under-representation of the region in genomic datasets [55, 56].

### Everything is everywhere: convergent ecology in divergent *E. coli*

**Figure 2:**
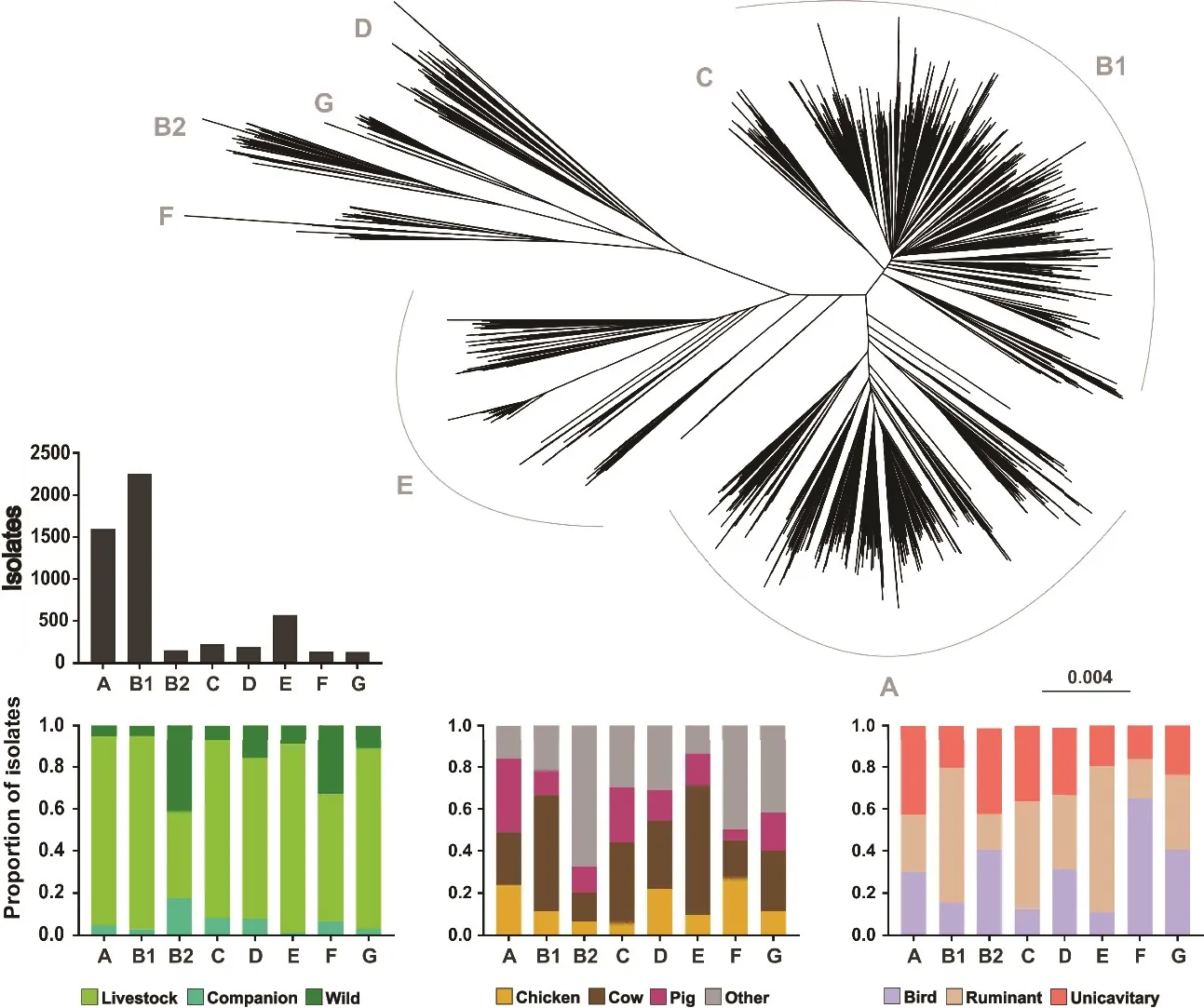
Phylogeny reveals that *E. coli* phylogroups do not segregate by host species, physiologies, or ecology. (A) NJ tree representing a MASH generated distance matrix of 5259 *E. coli* isolates. Bar charts show (B) the number of isolates per phylogroup and stacked bars with the proportion of isolates per phylogroup divided by niche categories: (C) ecology, (D) host species, (E) physiology.

Using a Neighbor-Joining (NJ) tree, constructed from a MASH-generated distance matrix, we identified eight distinct phylogenetic lineages corresponding to the established phylogroups A (*n* = 1595), B1 (*n* = 2255), B2 (*n* = 153), C (*n* = 225), D (*n* = 191), E (*n* = 572), F (*n* = 136) and G (*n* = 132) (Fig. 2A). Maximum-likelihood phylogenies of core genome alignments for individual phylogroups revealed a high degree of population structure, particularly in phylogroups A and B1. Importantly, all major phylogroups were represented in each of the niche categories investigated, suggesting a lack of consistent patterns linking deep-branching lineage structure with particular host species or ecology (Fig. 2C and D). Notwithstanding, some phylogroups were overrepresented in some niche categories. For instance, 56% (1248/2228) of cattle isolates belonged to phylogroup B1, whereas 41% (63/153) of phylogroup B2 isolates were sampled from wild sources, and 64% (87/136) of phylogroup F isolates were sampled from birds.

### Accessory genome variation underpins niche segregation

**Figure 3:**
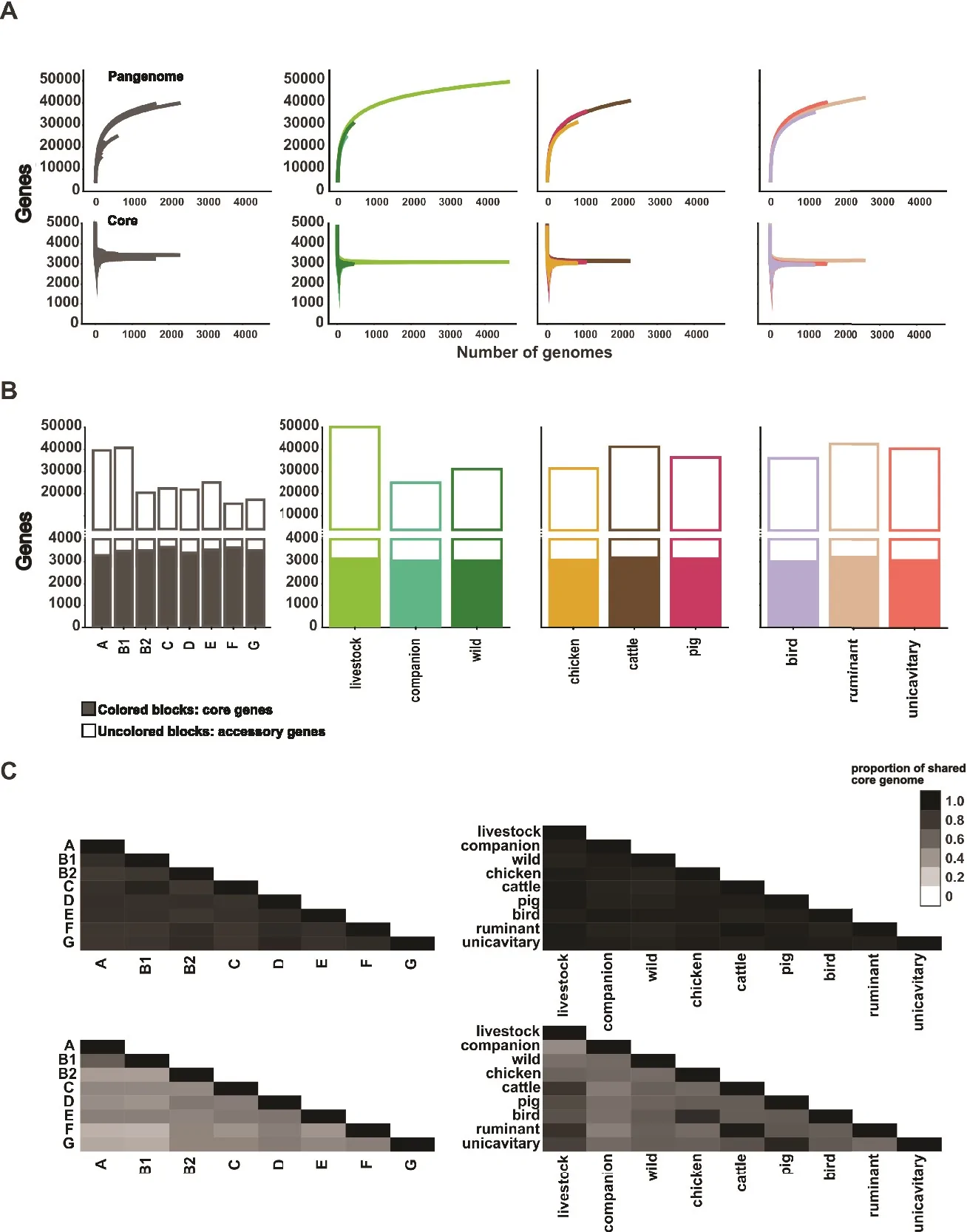
Pangenome variation between phylogroups and niche categories. (A) Pan-genome and core genome size relative to the number of isolates per phylogroup and niche category. (B) Bar chart showing the number of genes present in the core genome (solid fill) and accessory genome (no fill). (C) Matrix representing the number of core and accessory genes shared between (i) phylogroup and (ii) niche categories.

Pan- and core-genomes of all isolates were constructed based on COGs. The average number of genes per isolate was 4853 (SD 299). The total pangenome consisted of 51 205 COGs, with a core genome containing 3049 genes shared by >95% of isolates. In addition, we quantified the core and accessory genome for each phylogroup and niche category (Fig. 3A and B, Supplementary Table S2 & S3). In all cases we observed an open pangenome, as expected for *E. coli* [57, 58].

Phylogroups A and B1 have the largest pangenomes, correlating with their sample size, with 35 892 and 36 856 genes, respectively. Regarding the niche categories, livestock-derived isolates displayed the largest pangenome, encompassing 91% (46 456/51 205) of all genes, whereas isolates from companion sources had the smallest pangenome at 42% (21 385/51 205). The size of the core genome for each niche category differed by a maximum of 204 genes (range: 2964–3168). This level of consistency suggests that sampling was sufficient to capture general trends in core and accessory genome variation. To account for variations in phylogenetic distance from biased sampling, we used a model to predict the pan-genome size for a population size of 100 isolates per niche category [44] (Supplementary Table S4). Although prediction based on 100 isolates underestimated pangenome size, they followed the same trend as in the full dataset with the smallest and largest accessory genomes found in companion and livestock animals, respectively.

The core genome of *E. coli* isolates was remarkably similar between phylogroups or niche categories (Fig. 3C). Between 79% and 90% of core genes were conserved across phylogroups. Isolates from chickens, cattle, and pigs share up to 95% of their core genes. In contrast, the accessory genome varied considerably between phylogroups and niche categories. Isolates from different phylogroups shared some accessory genome content, ranging from 29% to 63%. The proportion of accessory genes shared by isolates from different niche categories ranged from 44% to 96%. Complementary niche categories, such as chicken and bird, were the most similar. For example, *E. coli* from ruminants and cattle shared 96% of their accessory genome, meaning that additional ruminant isolates (sheep and deer) only marginally contribute to the pangenome content already present in cattle isolates. It is expected that the number of accessory genes identified will relate to the number of isolates sampled. However, even if there is biased sampling, the shape of the gene discovery curve (Fig. 3A) provides information about the diversity of strains within a given niche. Our findings suggest some consistency in core genome content between *E. coli* derived from different sources, whereas variation in the accessory genome is strongly associated with host adaptation and niche segregation.

### Multiple parallel pangenome-wide association studies reveal ecological adaptations

**Figure 4:**
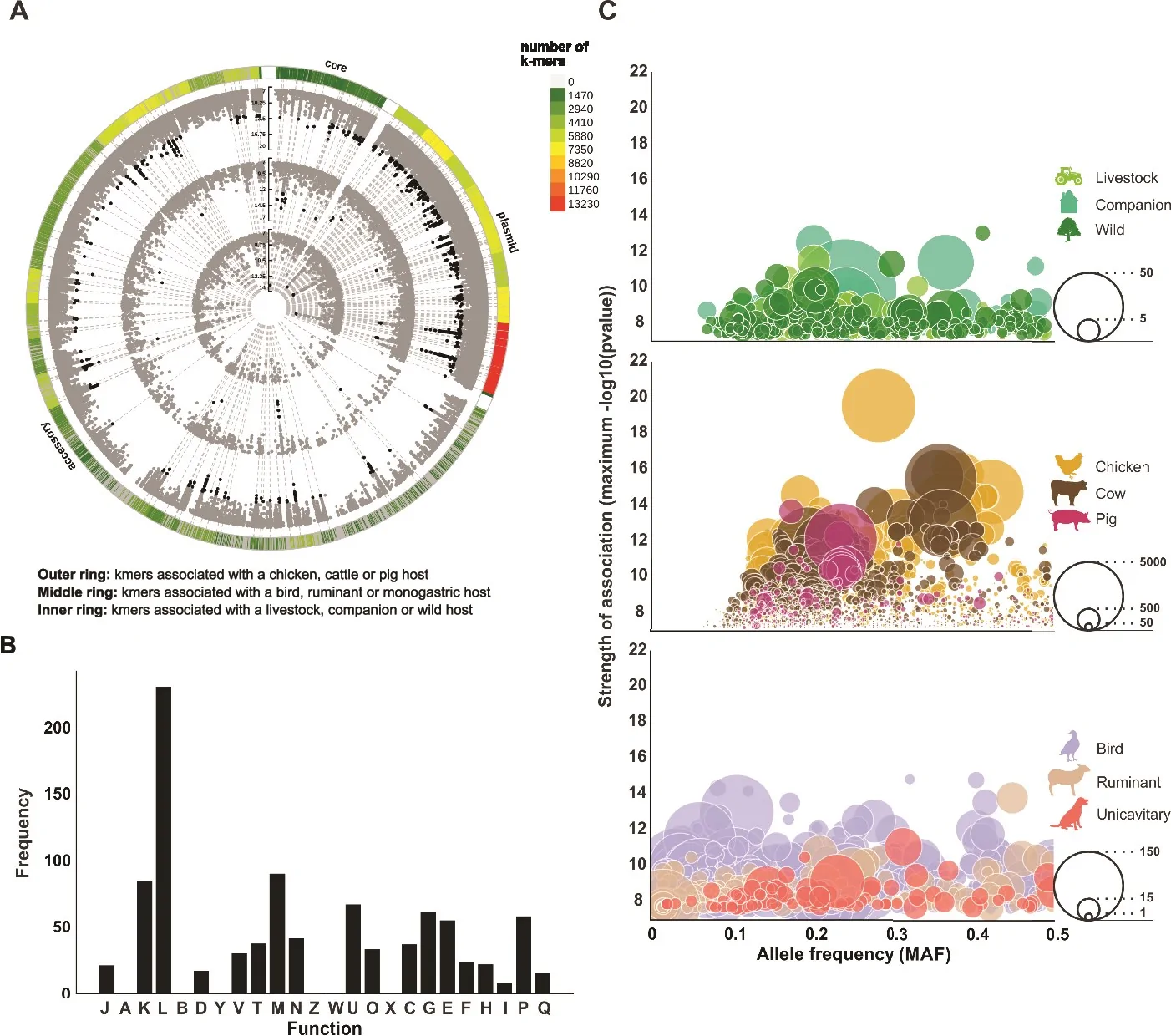
Multiple parallel pangenome-wide association studies reveal source adaptive sequence variation. (A) Circular–Manhattan plot of the pan-genome wide association mapping of significant *k*-mers associated with at least one niche category. The outer ring shows *k*-mers associated with a chicken, cattle, or pig host. The middle ring shows *k*-mers associated with a bird, ruminant, or monogastric host. Inner ring shows *k*-mers associated with a livestock, companion, or wild host. The *k*-mers within the 90th percentile of *P* values (3x10^−13^) are shown in black, whilst all other *k*-mers are grey. (B) Functional annotation of genes displaying niche associated genetic variation identified by GWAS. Functional categories include, J: Translation, ribosomal structure, and biogenesis; A: RNA processing and modification; K: Transcription; L: Replication, recombination and repair; B: Chromatin structure and dynamics; D: Cell cycle control, cell division, chromosome partitioning; Y: Nuclear structure; V: Defense mechanisms; T: Signal transduction mechanisms; M: Cell wall/membrane/envelope biogenesis; N: Cell motility; Z: Cytoskeleton; W: Extracellular structures; U: Intracellular trafficking, secretion, and vesicular transport; O: Posttranslational modification, protein turnover, chaperones; X: mobilome (prophages, transposons); C: Energy production and conversion; G: Carbohydrate transport and metabolism; E: Amino acid transport and metabolism; F: Nucleotide transport and metabolism; H: Coenzyme transport and metabolism; I: Lipid transport and metabolism; P: Inorganic ion transport and metabolism; Q: Secondary metabolites biosynthesis, transport and catabolism. (C) Bubble plots summarizing the *E. coli* genes and associated statistics for (i) ecological, (ii) host species, and (iii) physiological, niche categories. Bubble size represents the number of *k-*mers mapped to the gene.

A total of 100 pangenome-wise association studies were conducted in parallel for each of the nine niche categories to identify adaptive signatures in the genome (900 experiments). In every case, variable length unitig *k*-mers from the pangenome of 200 randomly sampled isolates were tested for their association with the niche of interest, and significance was determined using a Bonferroni corrected threshold of -log_10_(*P*) = 7.5, averaged across experiments. In total, 157 652 *k*-mers exceeded the threshold for significance (Fig. 4A; Supplementary Table S5, FigShare 10.6084/m9.figshare.30543260). Host species association experiments revealed the largest number of hits, with 25 558 significant *k*-mers mapping to 1726 unique genes, of which 1273, 1176, and 335 genes mapped to chickens, cattle, and pigs, respectively. In comparison, the physiology and ecology association experiments produced far fewer hits: 5311 and 1396 significant *k*-mers, which mapped to 1105 and 412 genes, respectively (Fig. 4C).

In pilot experiments, host species-associated genetic variation dominated over gut physiology categories. For example, 40% (6079/15 326) of bird gut associated genetic elements were previously identified by chicken GWAS experiments, and these variants had a greater association with chicken isolates than isolates from non-chicken birds. Therefore, to account for this host species dominance, chicken, cattle, and pig isolates were excluded from our gut physiology association experiments. The significant *k*-mers from all experiments were combined and tested for their niche category associations across the entire *E. coli* pangenome. From a total of 157 652 *k*-mers that exceeded the *P* value threshold of –log_10_(*P*) = 7.5, 20 011 *k*-mers had a specificity >60%, sensitivity >25%, and Cramer’s V >0.1, in at least one niche category. These niche-segregating *k*-mers included 12 687, 14 280, and 12 420 sequences associated with the species, physiology, and ecology categories, mapped to 1460, 1485, and 1445 genes, respectively.

A total of 1726 unique genes across the *E. coli* pangenome show variation associated with at least one niche category. This includes 5% (144/3049) of core genes, which account for 5% (907/20 011) of niche-segregating *k*-mers. Comparatively, just 3% (1582/48 156) of accessory genes show variation, but account for 70% (14 051/20 011) of niche-segregating *k*-mers, supporting accessory genome variation as the primary driver of niche association in *E. coli*. Furthermore, 58% (11 711/20 011) of niche-segregating *k*-mers mapped to plasmid sequences. This contrasts with comparable analyses in *Campylobacter* [59], *Helicobacter* [60], and *Staphylococcus* [61], whereby variation in chromosomal sequence is responsible for host associated adaptation. In *E. coli*, plasmids act as successful backbones for adaptation, such as promoting antibiotic resistance and enhancing bacterial competition, driving phenotypic evolution independently of the chromosomal genetic background [62].

### *Escherichia coli* isolates can be attributed to the correct source based upon host segregating genetic variation

**Figure 5:**
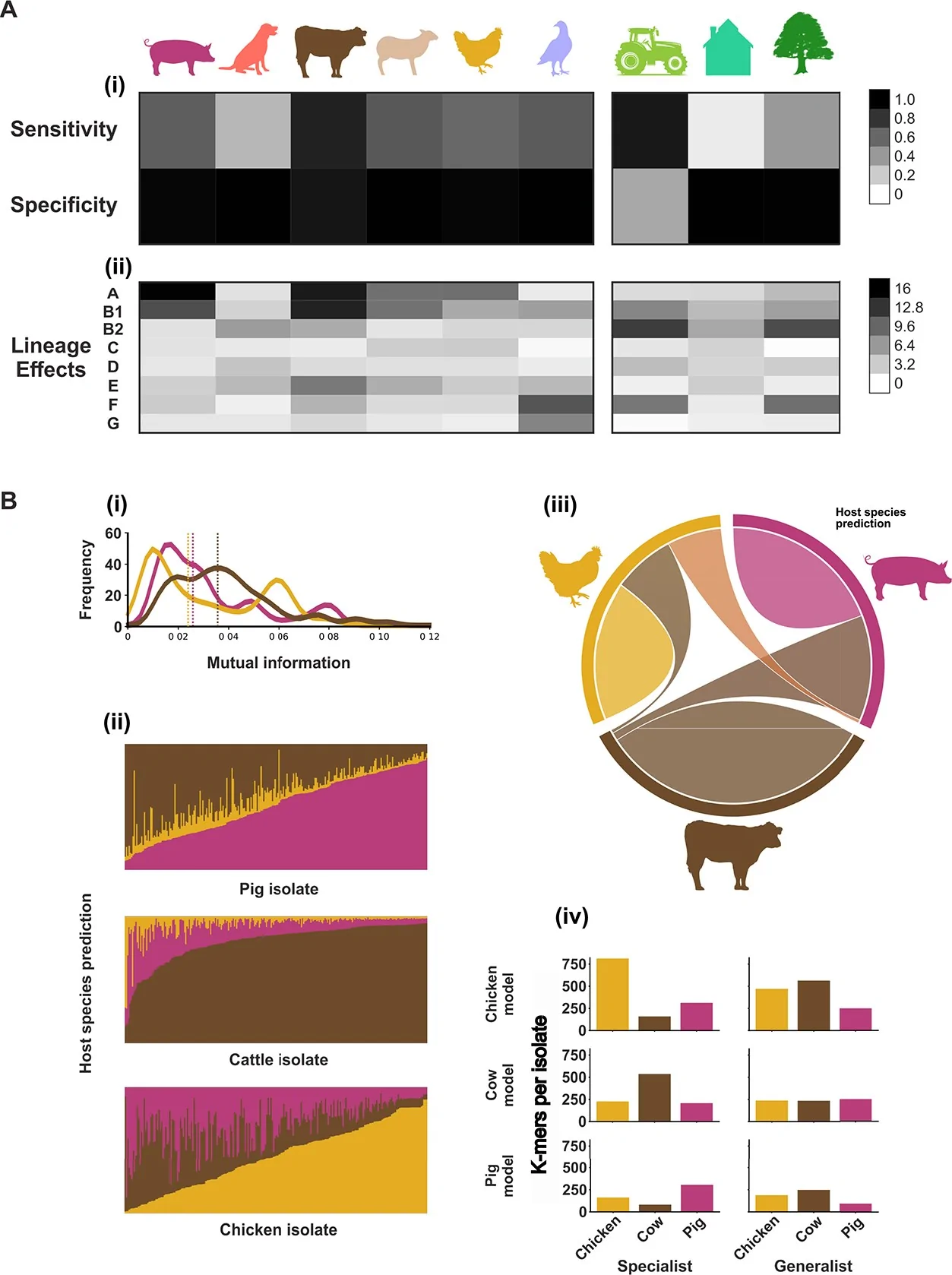
Elastic net regression model predictions of *E. coli* host niche. (A) Self-attribution of *E. coli* (i) sensitivity and specificity of niche models; (ii) lineage effects of niche models measured using Wald-test. (B) Validation source-attribution of *E. coli*: (i) mutual information between niche-segregating *k*-mers for chicken, cattle, and pig niches; (ii) host species predictions made by niche models for isolates sourced from chicken, cattle, and pig isolates; (iii) results from niche model predictions visualized as a chord diagram. Each host niche is represented by a fragment on the outer part of the circular layout. The size of the arcs connecting each niche is proportional to the number of isolates that have been assigned to a host niche, either correctly or incorrectly; (iv) *E. coli* isolates were divided into either specialist (those that were correctly attributed to their host niche) or generalist (those that were incorrectly assigned to a different host niche) isolates. Bars indicate the average number of niche-segregating *k*-mers shared between each host niche model and specialist/generalist *E. coli* from each niche.

The degree to which source associated genetic variation segregates is a measure of genetic cohesion allowing isolates to be attributed to a source population. Here, we use elastic net linear regression machine learning models to attribute *E. coli* isolates to their source based on the presence of niche-segregating *k*-mers. Our curated dataset of 5259 *E. coli* was randomly partitioned into two datasets, one containing 75% of isolates (the training dataset), and the other containing 25% of isolates (the test dataset). For each niche category, the distribution of niche-segregating *k*-mers across the training dataset was used to construct a source-attribution model using the *--wg* option in Pyseer [49]. The accuracy of each model was evaluated by comparing the model’s niche predictions for the test dataset with their true sample origins. This self-attribution was conducted for the three levels of niche organization (host, physiology, and ecology) (Fig. 5A).

A total of 75% (772/1020) of *E. coli* isolates were correctly assigned to their host source (chicken, cattle, or pig). Model sensitivity was calculated as the number of true positives divided by the sum of true positives and false negatives, multiplied by 100 to express it as a percentage. Specificity was calculated as the number of true negatives divided by the sum of true negatives and false positives, also expressed as a percentage. The cattle model had the highest sensitivity (87%; 486/557) but lower specificity (92%; 694/756) than the chicken and pig models (98% specificity; 1089/1111 and 1030/1052, respectively). This likely reflects the prevalence of cattle-associated genetic variants in non-cattle sources. Self-attribution based upon physiology correctly classified 65% of bird (60/93) and ruminant (55/84), but only 28% (33/116) of monogastric isolates (Supplementary Fig. S1). The model’s ability to identify *E. coli* isolated from chicken, cattle, or pig sources was low—21% (43/202), 10% (58/557), and 3% (9/261), respectively. Applying host models to corresponding physiological groups gave true-positive rates of 27% (25/93) for birds, 19% (16/84) for ruminants, and 6% (7/116) for monogastric animals. Self-attribution based upon ecologically segregating *k*-mers assigned 98% (1050/1061) of livestock isolates correctly, but just 40% (41/102) of wild and 8% (4/50) of companion animal isolates. Companion isolates shared more genomic variation with livestock (82%; 41/50) than wild sources (58%; 59/102).

Variation within phylogroups significantly influenced model predictions (Wald test), with lineage effects reflecting niche distribution (Fig. 5A). Livestock and wild models showed nearly identical lineage patterns, but the strongest ecological signals appeared in phylogroups B2 and F, suggesting wild-derived variation is most informative. Mutual information analysis (Fig. 5B) further revealed that *k*-mer patterns associated with the cattle niche exhibited the highest discriminatory power, indicating a greater dependence of genomic variation on cattle-associated *E. coli* compared to those from chicken or pig sources.

### Model validation revealed robust genomic host associated genomic variation

To validate predictive ability, we tested host models on an independent set of 722 *E. coli* isolates. Host source was assigned based on the highest probability across chicken, cattle, and pig models. Overall accuracy was 71% (513/722), with greatest sensitivity in the cattle model at 96% (246/256), followed by pig at 60% (144/240), and chicken at 54% (123/226) (Fig. 5B). As in training, the cattle model showed the lowest specificity at 68% (318/466), misclassifying 55 chicken and 93 pig isolates. The pig model also misassigned 55 isolates, mostly from chickens. The chicken model had the highest specificity at 98% (460/466), misclassifying only six isolates.

To further understand variation in prediction accuracy, isolates were classified as “specialists”, where source was confidently determined based on the distribution of niche-segregating *k*-mers, and generalists where it cannot (Fig. 5B). Specialists shared more segregating *k*-mers with their respective host model than generalists. In contrast, generalist isolates from different host species shared similar, lower numbers of model *k*-mers. This pattern was consistent across all three models. Finally, mapping predictions onto a maximum-likelihood phylogeny (Supplementary Fig. S2) showed generalists were not confined to specific lineages but scattered across the tree. This suggests misclassifications stem from the genomic similarity of generalist isolates to non-source host profiles, rather than poor predictive power within specific lineages. Instead, the lower *k*-mer sharing and broader distribution of generalists may reflect recent host transitions or inherent flexibility in niche adaptation.

### Ecological and physiological adaptations dominated in birds and ruminants respectively

**Figure 6:**
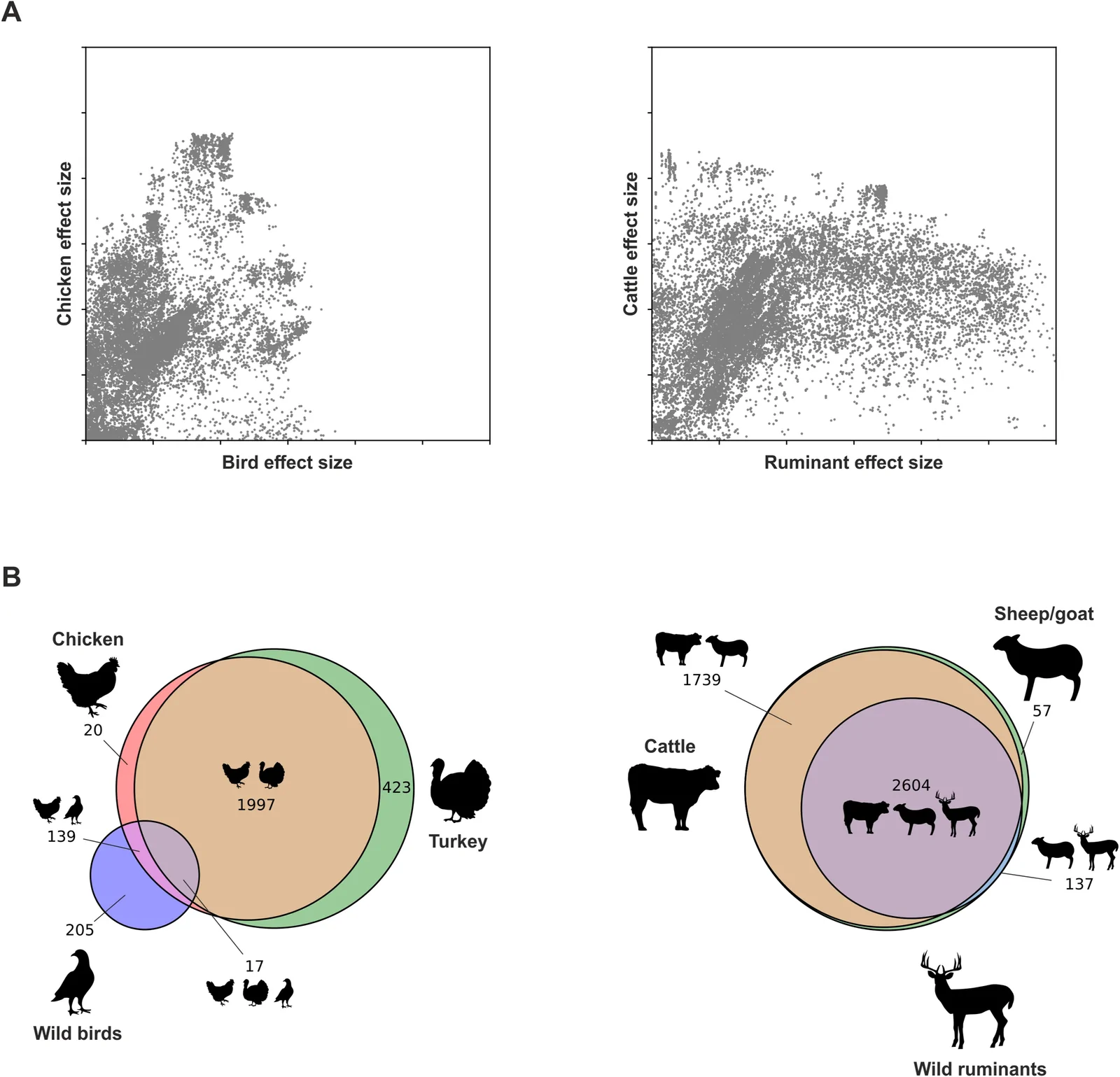
Sequence variation is shared between *E. coli* with the same host physiology. (A) Scatter plot comparing the effect size of niche-segregating *k*-mers between host and host physiology niche categories. (B) Venn diagram depicting the number of bird or ruminant-segregating variants associated with each niche category.

As shown in our self-attribution experiments, sufficient genetic variation exists to classify *E. coli* isolates to bird and ruminant physiological niches. To explore the nature of these adaptations, we examined the distribution of niche-segregating *k*-mers using Cramer’s V to quantify association strength between variants and host categories (Fig. 6). Comparing *k*-mer effect sizes between physiological (bird or ruminant) and ecological (livestock or wild) niches reveals distinct patterns of adaptation. In birds, most *k*-mers show significant associations with both bird and chicken niches (Fig. 6A). However, many of these *k*-mers are strongly associated with chickens but only weakly with birds, whereas the reverse is rare. This asymmetry suggests that adaptation to birds is dominated by ecological traits specific to chickens, rather than avian physiology. In contrast, most *k*-mers associated with the cattle niche also show strong associations with the broader ruminant niche, supporting the idea that *E. coli* adaptation to ruminants is driven primarily by host physiology.

Further evidence comes from comparing bird-associated variants across species (Fig. 6B). Of 2173 *k*-mers associated with chickens, 92% (1997/2173) are shared with domestic turkeys but only 6% (139/2173) with wild birds. Similarly, 57% (205/361) of wild bird-associated *k*-mers are not found in domestic birds, reinforcing the dominant role of shared livestock ecology (chicken and turkey), rather than physiology in driving adaptation. Contrastingly in ruminants, 4343, 4400, and 2741 ruminant-segregating *k*-mers were associated with cattle, sheep/goat, and deer, respectively, with 57% (2604/4537) shared across all three. This widespread overlap across wild and domestic hosts suggests that physiology, rather than ecology, is the principal driver of adaptation in ruminants.

## Discussion

Evolutionary theory predicts that organisms in isolated niches will diversify from the ancestral population, giving rise to niche associated lineages, and ultimately new species. Where multiple lineages occupy the same niche, they will compete, and one ecotype will prevail [8, 63]. Based on this model, one would expect to observe niche associated *E. coli* lineages. However, consistent with previous work, we found that all major *E. coli* phylogroups were observed in all niche categories investigated here. Therefore, it may be reasonable to conclude that “everything is everywhere”, at least at the level of phylogroup. This may seem to contract the ecotype model but there are several explanations for the ubiquity of phylogroups. First, there may be multiple non-competing sub-lineages within the phylogroups that occupy different subniches within the host gut [64]. Second, continuous niche transitions, within and between hosts, may give rise to a dynamic system with ongoing colonization events, disrupting direct lineage competition [65]. Third, sub-lineages may adapt by horizontally acquiring niche specifying genes before they are outcompeted [28, 66].

The absence of clear host-associated lineage structure is consistent with relatively recent, and ongoing, host-transition. When bacteria transition between hosts they adapt to the new niche, and, importantly, the most beneficial adaptations will increase most rapidly in the pioneer population [67]. This fundamental principal, that guided the first formal bacterial GWAS [59], means that the most strongly niche-associated genetic elements will likely be linked to adaptation driven by selection, rather than genetic variation that has evolved through drift in allopatry. One may expect *E. coli*, adaptation to be dominated by the acquisition of mobile genetic elements [28, 68], particularly as plasmids enable the simultaneous acquisition of multiple beneficial traits. However, as putative plasmid genes were defined by reference to a database using the annotation tool [52], only *k*-mers matching previously described plasmids were identified as such. This may lead to underestimation of plasmid genes. We also identified a number of phage integrase and transposon machinery genes associated with specific hosts, which could further drive rapid transfer of genetic material. This “plug and play” ability could explain how divergent lineages colonize the same host niche. However, although numerous *k*-mers did map to plasmid borne genes, our pangenome-wide association studies revealed the importance of niche-specifying alleles, rather than just simple gene presence, consistent with a mosaic of core and accessory genome elements driving niche adaptation.

Parallelized pangenome-wide association studies (900 *in silico* experiments) revealed genomic variation linked to all nine niche categories. The putative function of genes with host segregating variation give clues about the specific adaptations to that host or niche. There were a total of 16 984 chicken associated *k*-mers that mapped to 58 genes. The most significant variation was detected in *icsA*, encoding a surface protein that facilitates intra/intercellular motility of *E. coli* by nucleating actin filaments at one pole of the bacterium to form an “actin rocket”, a phenotype associated with entero-invasive *E. coli*. IcsA also functions as an adhesin which promotes invasion into host epithelial cells [69, 70]. Other invasion effector genes, such as the proteases *sepA, hbp, espP*, and *ompT* were also associated with the chicken niche. Multiple toxin-antitoxin (TA) systems were associated with chicken adaptation, including *relBE, mazEF*, and *yafNO*, which inhibit protein synthesis in response to stress [71–73]. The *CbeA-CbtA* TA system, involved in cytoskeletal remodeling and antibiotic resistance, was also chicken-associated. Together, variation in these virulence and TA genes suggests chicken adaptation involves altered invasion and stress response phenotypes.

Host diet may also be an important factor for gut colonization. Chicken associated genetic variation was observed in the plasmid-borne *raf* operon, which enables uptake of raffinose, a non-digestible galacto-oligosaccharide that can constitute up to 10% of soybean meal, the primary protein source in poultry feed, and impacts the chicken gut microbiota [74]. *lacY*, encoding lactose permease, had similar variation [75].These genes indicate adaptation to diet, but chicken associated genetic variation was also linked to AMR. Specifically, the *fosA* and *mcr-1* genes, linked to fosfomycin and colistin resistance respectively [76, 77], were present in ~20% of all chicken *E. coli* isolates compared to 4%–8% from other sources. Although the isolates in our study are not from a structured survey, these findings are consistent with other studies [78] and may be related to the use of these antimicrobials to treat enteric infections in broiler chickens. For these host-associated genes, plasmid carriage may enhance colonization success in addition to facilitating resistance.

In *E. coli* isolated from cattle, many of the most significantly host associated genes were linked with virulence and the Shiga toxin-producing strain of *E. coli* O175 that causes severe foodborne infections in humans. These included: the *hyl* locus, encoding α-hemolysin, which lyses erythrocytes and is common in invasive strains [79, 80]; *shlB* and *hbp*, linked to red blood cell lysis and intra-abdominal infections; *sepA*, derived from *Shigella* and co-associated with cattle associated hemolysins [81, 82]; *espP*, encoding an extracellular serine protease essential for cattle colonization by *E. coli* O157:H7 [83]. This suggests *E. coli* adapted to the cattle niche have a modified ability to acquire iron and other nutrients from erythrocytes.

In comparison to chickens and cattle, genetic variation associated with pigs lacked putative virulence and toxin genes but included several AMR genes. These included *bcr, bla*, and *tetR*, linked to bacitracin, beta-lactam, and tetracycline resistance, respectively. The strongest pig associated variation was in the *sil, cus*, and *cop* operons, which confer resistance to silver and copper. These metals are widely used as biocides in veterinary settings, with copper specifically used as a growth promoter in pig farming [84, 85]. Exposure has resulted in resistant *E. coli* via inducible efflux systems such as *sil* and *cus* [86–89]. This is particularly problematic as metal and antibiotic resistance genes often co-occur on plasmids. Therefore, exposure to metals may co-select for AMR, as seen with dietary zinc [90] and the *sil* genes, which are over-represented in extended-spectrum beta-lactamase producing *E. coli* [91]. Taken together, resistance to antimicrobial metals appears to be an important factor in adaptation to the pig niche and may bring the risk of co-acquired antibiotic resistance.

Specific phenotypic adaptations to particular hosts give rise to genetic variation that consequently segregate by host. These are the adaptive genomic signatures that are flagged by GWAS, but this genetic variation can also be used to attribute the origin of particular strains [92–95]. This simple principal underlies various source attribution models [96, 97] but the degree of host segregation is also informative for understanding population structure. The ability to attribute *E. coli* isolates to their correct host niche varied with some “specialist” *E. coli* genotypes showing evidence of specific adaptations and host segregation whereas other genotypes appeared to have host generalist ecology, indicating a greater propensity for host transition. Identifying significant host segregating *k*-mers was less likely among putative generalists for multiple reasons. First, *k*-mers with low *P* values in relation presence/absence are excluded in the elastic net linear regression model where they do not add values to improve model performance (i.e. redundancy due to multicollinearity). Second, *k*-mers that are adapted to an unsampled host or were transiently present in their non-preferred hosts at the point of sampling will be wrongly associated with this host. Both of these observations imply that there is no absolute physical barrier to host switching, consistent with the “everything is everywhere” aphorism.

Nonetheless, host physiology and ecology do represent important barriers to colonization. We found clear evidence that *E. coli* adapt to the ruminant gut in the same way in both domestic and wild hosts, with cattle, sheep, goat, and deer all sharing a high proportion of ruminant-associated *k*-mers, despite their diverse ecology (Fig. 6B). Conversely, among *E. coli* isolated form birds, host ecology was a stronger predictor, with domesticated birds sharing more associated *k*-mers than with their wild counterparts, despite their common physiology. Although wild animals have diverse ecologies, livestock typically share ecological traits including high stocking density, a consistent diet, low genetic variation, and exposure to antimicrobials. This complexity in host natural history may explain why *E. coli* adaptations can be driven by both host physiology and ecology.

In conclusion, we describe an ecological landscape for *E. coli* that involves ongoing host transition, consistent with traditional explanations of bacterial population biology [4]. Successful colonization is associated with a mosaic of adaptions across the entire pangenome. The hierarchical GWAS approach identified candidate adaptive genes at three levels of organization, demonstrating the advantage over a reductive single-level comparison. This approach was made possible through the utilization of publicly available genomes, ensuring a sample size large enough to identify robust associations. It is acknowledged that the underlying sampling bias cannot be excluded as public *E. coli* genomes are often sampled in the context of specific projects or outbreak investigations. However, our ecological genomics approach provides valuable high-level information about the evolutionary forces that shape natural *E. coli* populations and zoonotic bacteria more generally. This addresses long-standing questions about bacterial biogeography, but also provides a quantitative basis for considering the transmission of zoonotic bacteria that is essential for improved animal welfare and food safety.

## Supplementary Material

wraf267_Supplemental_Files

## Data Availability

The datasets analyzed during the current study are available in the FigShare repository, https://doi.org/10.6084/m9.figshare.30543260.
