# Supplementary material for: Everything is everywhere but Escherichia coli adapts to different niches
Source: ISME J. 2025 Dec 18;20(1):wraf267. doi: 10.1093/ismejo/wraf267 (PMC12815263; doi:10.1093/ismejo/wraf267)
Supplement: wraf267_Supplemental_Files [file wraf267_supplemental_files.zip › Supp_Fig_2_wraf267.pdf]

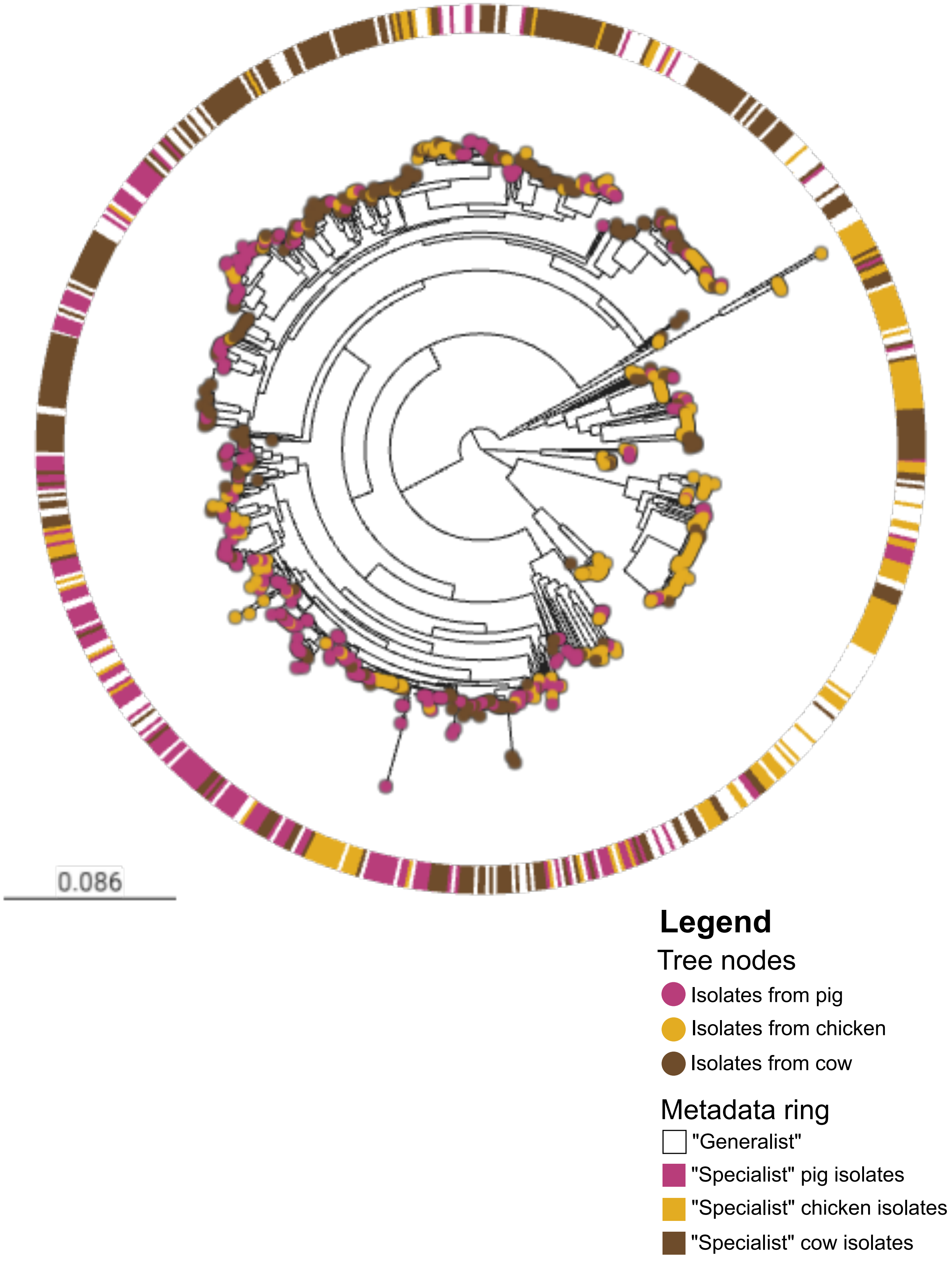
0.086
Legend
Tree nodes
Isolates from pig
Isolates from chicken
Isolates from cow
Metadata ring
"Generalist"
"Specialist" pig isolates
"Specialist" chicken isolates
"Specialist" cow isolates
