# Supplementary figures and images for: Everything is everywhere but Escherichia coli adapts to different niches
Source: ISME J. 2025 Dec 18;20(1):wraf267. doi: 10.1093/ismejo/wraf267 (PMC12815263; doi:10.1093/ismejo/wraf267)

**A**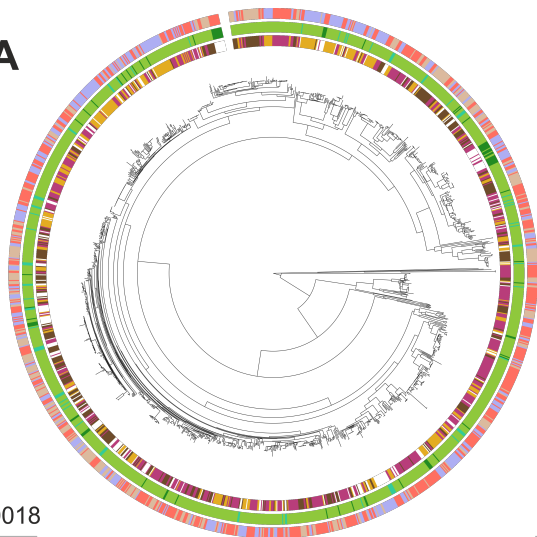**B1**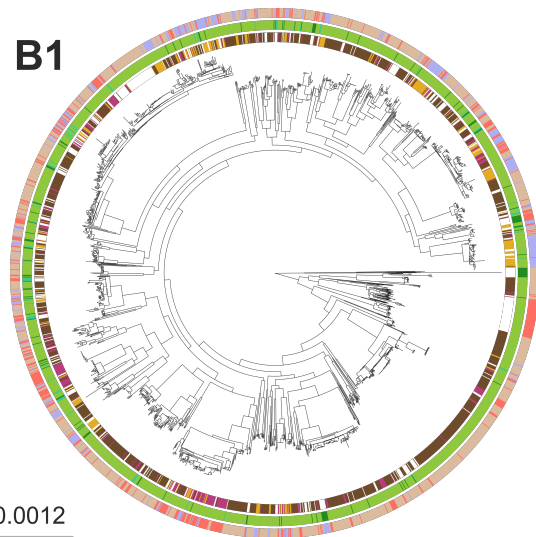**B2**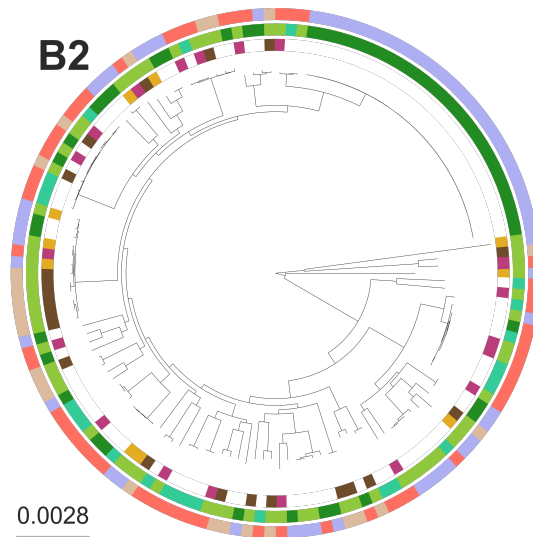**C**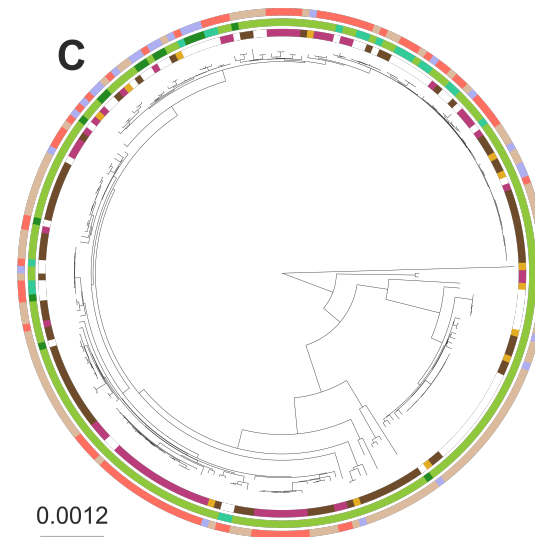**D**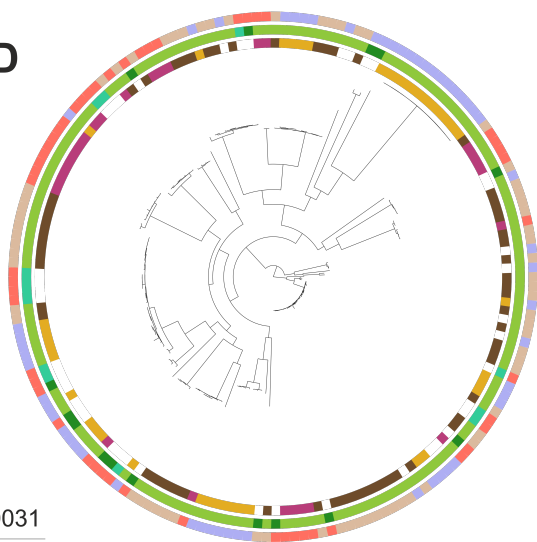**E**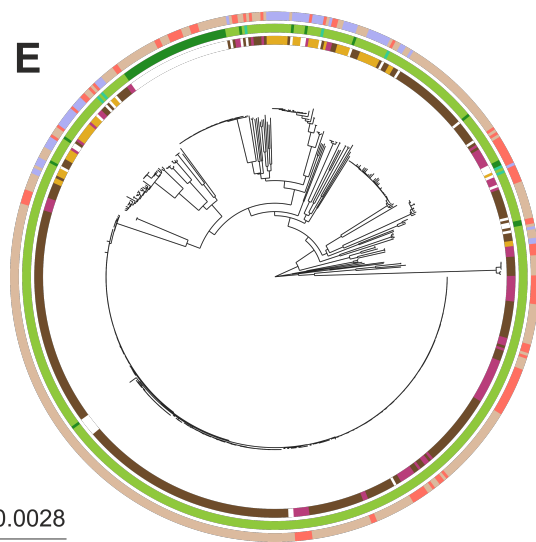**F**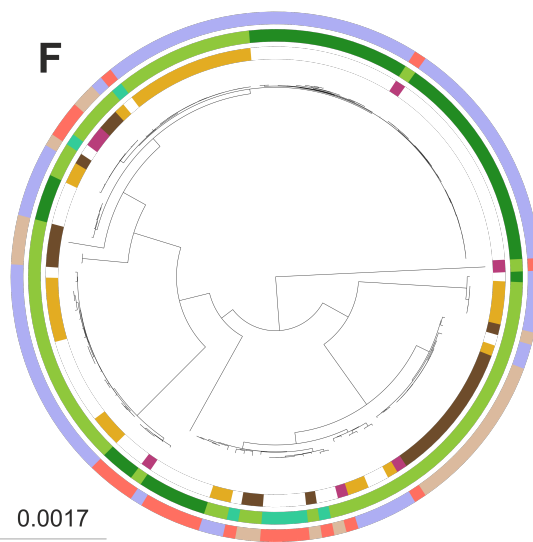**G**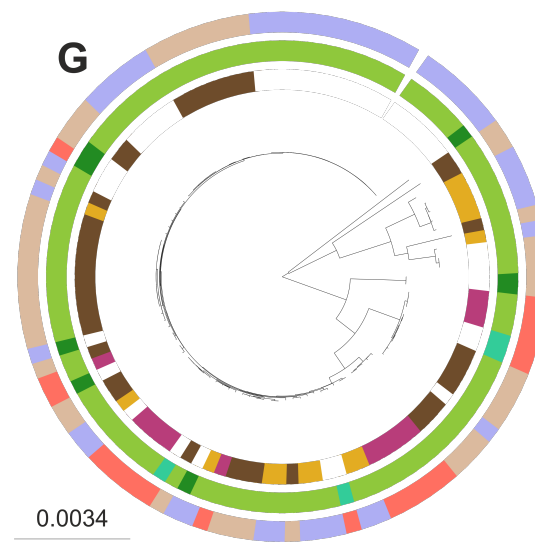

Supplement: wraf267_Supplemental_Files [file wraf267_supplemental_files.zip › Supp_Fig_1_wraf267.pdf]
